# A qualitative exploration of reunification post alienation from the perspective of adult alienated children and targeted parents

**DOI:** 10.3389/fpsyg.2023.1189840

**Published:** 2023-08-03

**Authors:** Mandy Louise Matthewson, Jessica Bowring, Jacinta Hickey, Sophie Ward, Peta Diercke, Leesa Van Niekerk

**Affiliations:** ^1^School of Psychological Sciences, College of Health and Medicine, University of Tasmania, Hobart, TAS, Australia; ^2^School of Education, The University of Newcastle, Callaghan, NSW, Australia

**Keywords:** parental alienation (PA), parental alienating behaviors, targeted parents, alienated child/children, trauma informed approach

## Abstract

**Introduction:**

The aim of this study was to explore the experiences of voluntary reunification from the perspectives of adult alienated children and targeted parents

**Methods:**

Nine adult alienated children and 12 targeted parents participated in semi-structured interviews which were transcribed verbatim and analyzed thematically

**Results:**

Six themes emerged in the data from the adult alienated children including catalysts for reunification, factors influencing reunification, adult alienated child relationships, the role of communication in reunification, adult alienated child understandings of alienation post reunification, and the role of therapy in reunification. Three themes emerged from the targeted parents including what is reunification, factors impacting reunification, and life after reunification.

**Discussion:**

Findings from the present study offer novel insights into the experience of voluntary reunification from the perspectives of both adult alienated children and targeted parents. They illustrate that voluntary reunification is a process that takes time. This process can span decades and can include periods of connection and rejection.

## What is parental alienation?

Parental alienation occurs when a child strongly aligns with one parent (the alienating parent), while rejecting a relationship with their other previously loved parent (the targeted parent) (Bernet et al., 2010). The rejection of the targeted parent is a consequence of parental alienating behaviors used consistently by the alienating parent to undermine the targeted parent–child relationship (Haines et al., 2020). Parental alienation commonly occurs in the context of separation and/or divorce.

The alienated child’s relationship with the targeted parent is often characterized by withdrawal, hostility, or contempt (Clawar and Rivlin, 2013). It is suggested that the attachment between the alienated child and targeted parent is distorted because of the alienating parent delivering inaccurate messages to the alienated child about the targeted parent in conjunction with interruptions to time the child spends with the targeted parent. Such messages and an inability to spend continuous time with the targeted parent over-ride the alienated child’s actual history and experience of security with the targeted parent (Baker, 2007; Harman et al., 2021). The primary outcome of being exposed to parental alienating behaviors is the complete breakdown and loss of relationship between a parent and child (Baker, 2007; Bentley and Matthewson, 2020; Harman et al., 2021; Verhaar et al., 2022).

Alienating parents may differ vastly in the type and number of parental alienating behaviors they use. Common parental alienating behaviors include emotional manipulation of the child and targeted parent, disrupting time the child spends with the targeted parent, withholding or interfering with information the targeted parent receives about their child, encouraging the child to defy the targeted parent, defaming/denigrating the targeted parent to the child and in front of the child, and erasing the targeted parent from their child’s life (Haines et al., 2020; Harman and Matthewson, 2020).

### Parental alienating behaviors as family violence

Parental alienating behaviors are widely considered a form of family violence in the literature (e.g., Bentley and Matthewson, 2020; Haines et al., 2020; Harman and Matthewson, 2020; Lee-Maturana et al., 2020; Verhaar et al., 2022). Family violence is defined as any act of violence perpetrated by one family member against another. It includes violence perpetrated by cohabitating/non-cohabitating partners/ex-partners, intimate partner violence (IPV) and the abuse and neglect of children (Chalk and King, 1998). A central feature of family violence is coercive control. Coercive control is a pervasive pattern of behaviors used by a family member to maintain power over and limit the autonomy and choices of another family member. It involves isolating victim/survivors from other family members, their children and friends, controlling access to resources, finances and regulating the victim/survivor’s daily life (Candela, 2016). Arguably, coercive control is at the heart of parental alienating behaviors.

### Impacts of parental alienating behaviors

The impacts of parental alienating behaviors are significant and severe. Parental alienating behaviors can create reality distortions and compromised reality-testing for alienated children. Parental alienating behaviors inhibit children’s interpersonal development to the extent that they can become socially withdrawn and have difficulties in relationships with others. These effects are often enduring (Baker, 2007; Bentley and Matthewson, 2020; Haines et al., 2020; Verhaar et al., 2022). Adult alienated children have reported experiencing depression, anxiety, low self-esteem, substance abuse, and trauma reactions in response to being alienated from a parent (Baker, 2007; Baker and Ben-Ami, 2011; Ben-Ami and Baker, 2012; Bentley and Matthewson, 2020; Verhaar et al., 2022). Adult alienated children also have described experiencing guilt and shame, mistrust in themselves and of others (Baker, 2007; Bentley and Matthewson, 2020; Verhaar et al., 2022). Further, adult alienated children can become alienated from their own children (Verhaar et al., 2022). Parents alienated from their own children due to parental alienating behaviors report experiencing moderate to high levels of depression, anxiety, stress (Vassiliou and Cartwright, 2001; Baker, 2010b; Balmer et al., 2018); social isolation, feelings of powerlessness, loneliness, despair, and suicidal ideation (Vassiliou and Cartwright, 2001; Balmer et al., 2018; Poustie et al., 2018; Lee-Maturana et al., 2020).

The effects of parent alienating behaviors and parental alienation can be understood using attachment theory. Bowlby stipulated that continuous, warm, and involved contact between a child and caregiver is necessary to form and sustain a healthy attachment. If any of these conditions are not met, an attachment, particularly a secure base, cannot be formed (Bowlby, 1988). Alienated children are led to believe through parental alienating behaviors that the targeted parent does not want or love them. The child’s critical thinking skills are compromised and their capacity to trust their own perceptions are damaged by this process, which results in internal working models of the self as unlovable and others as unsafe and unloving (Harman and Lorandos, 2021). This negatively impedes psychosocial development and preservation of a secure attachment between the child and the targeted parent. Several studies confirm this and demonstrate that adult alienated children are less likely to be securely attached in their adult relationships (Baker, 2007; Bentley and Matthewson, 2020; Harman et al., 2021; Verhaar et al., 2022).

### Voluntary reunification

Reunification between the targeted parent and child following parental alienation can occur. Reunification can occur because of therapeutic and legal interventions (see Templer et al., 2017). Voluntary reunification (also referred to as spontaneous reunification) can be defined as a process that occurs when an alienated child actively seeks to restore the relationship with the targeted parent independently from court orders or therapy (Darnall and Steinberg, 2008b).

There is limited research exploring voluntary reunification. Research to date has focused on catalysts for voluntary reunification. Baker (2007) explored the experience of adult alienated children and identified 11 catalysts for reunification. This included maturation of the child that allowed for the cognitive capacity to reflect on their childhood experience more objectively. The alienating parent turning on the child. Experiencing parental alienation as a parent. The targeted parent coming back into the child’s life thus giving the child experiences with the targeted parent that challenge the alienating parent’s narrative. Attaining a milestone that triggered a need to reconnect with the targeted parent. Engaging in therapy that was a safe and non-judgmental space facilitating reflection. Intervention of extended family. Intervention of significant others. Seeing the alienating parent mistreat others. Discovering that the alienating parent was dishonest. Becoming a parent. Voluntary reunification can also be prompted by a crisis or significant change the adult alienated child’s life (Rand and Rand, 2006; Darnall and Steinberg, 2008a,b).

Rand and Rand (2006), Baker (2007), and Darnall and Steinberg (2008a,b) highlighted the difficulties associated with defining successful voluntary reunification because the process is fragile and not a linear one. Successful voluntary reunification is achieved when there is a resumption of an ongoing and healthy relationship between an alienated child and both parents. According to this definition, one third of voluntary reunifications were considered successful in Darnall and Steinberg (2008a) study. Darnall and Steinberg (2008a,b) noted that successful voluntary reunification could partly be attributed to targeted parents resisting any desire to convince the child that the child’s interpretations or recollections of past events were wrong. Factors that limited successful voluntary reunification included targeted parents not meeting their child’s expectations and/or lacking empathy and emotional availability (Darnall and Steinberg, 2008a,b).

Baker and Fine (2014) examined the experience of targeted parents who had reunified with their children. They found that reunification was possible when the targeted parent never gave up hope of reunification, could see the alienation experience from the child’s perspective, had realistic expectations of the child and moved at the child’s pace. This was made possible by the targeted parents becoming educated and informed on parental alienation.

With limited research into the process of voluntary reunification, the aim of the current study was to examine the experience of both adult alienated children and targeted parents who have voluntarily reunified with a parent and targeted parents who have voluntarily reunified with their child/children. Currently, there are a lack of intervention programs designed to support families through the voluntary reunification process (Haines et al., 2020). The results of this research may help to inform recommendations for intervention programs.

## Methods

### Participants

The study included 9 participants identified as targeted parents who had reunified with at least one child; 7 participants identified as adult alienated children who had reunified with their targeted parents (6 of whom were currently alienated from their own children); and 2 participants identified as reunified targeted parents and reunified adult alienated child. In total, 11 targeted parents provided their stories of reunifying with a child and 9 adult alienated children provided their stories of reunifying with a parent.

Adult alienated children were 41–61 years of age. Three were from Australia and 6 were from the United States of America. All adult alienated children identified as female. Two had reunified with their mother and 7 had reunified with their father. Adult alienated children were reportedly alienated from a parent for 7–19 years. Some adult alienated children found it difficult to quantify how long they had been reunified for. This ranged from “not fully reunified” to over 20 years reunified. In some cases, adult alienated children said that although they had been in contact with their targeted parent for several decades, they did not consider their relationship with them to be fully reunified.

Targeted parents were 47–65 years of age. Seven were from Australia, 1 from Thailand, 1 from the United States of America, 1 from Canada and 1 from South Africa. Eight identified as female and 3 identified as male. Targeted parents were reportedly alienated from 1 to 3 children and reunified with 1–3 children. In most cases, targeted parents had not reunified with all their children. Targeted parents were alienated from their children from between 2 and 15 years. Targeted parents found it difficult to quantify how long they had been reunified with their children. The estimated length of time since reunification ranged from 2 weeks to 4 years.

### Procedure and materials

Ethics approval was obtained from the Social Sciences Human Research Ethics Committee at the University of Tasmania. Recruitment for the study was carried out nationally and internationally via advertisements on social media, online support groups for people affected by parental alienation and advertisements in psychology practices. The advertisement consisted of the following information: “*Were you isolated from one of your parents when you were younger as a result of conflict between your parents? Were you isolated from your child as a result of conflict with your partner or ex-partner? Have you since re-established a relationship with the parent or child you lost contact with? This study is looking at the process of reunification between alienated parents and their adult children*.” Interested individuals were invited to email the research team. They were then sent an information letter on the purpose and nature of the study, a consent form, and screening questionnaire. The targeted parent screening questionnaire was originally developed and employed by Balmer et al. (2018) and asked individuals to indicate if their child had been exposed to 13 common parental alienating behaviors. Individuals who endorsed at least six out of 13 items were considered eligible to participate. Adult alienated children were asked to complete the Baker Strategies Questionnaire (BSQ) (Baker and Chambers, 2011) and considered eligible to participate if they reported experiencing over 50% of noted parental alienating behaviors. All individuals were identified as having reunified post alienation.

Eligible individuals shared their experience of parental alienation and subsequent reunification with researchers in 60–90-min semi-structured qualitative interviews. Interviews were conducted via Zoom or Skype, both considered satisfactory and viable tools to use for the collection of qualitative data (Drabble et al., 2016; Lo Iacono et al., 2016; Archibald et al., 2019). Interviews were audio-recorded for accuracy and transcribed verbatim. Participants were given the opportunity to edit their transcripts to maximize data accuracy and clarity. Data analysis included only the edited transcripts.

### Data analysis

The current study followed a qualitative descriptive design. The aim of a qualitative descriptive design is to offer a summary of events (experienced by individuals or groups of individuals) in everyday language (Sandelowski, 2000). This type of design allows for the description of data in a manner that is low-inference and fact-based.

An inductive approach was utilized to analyze the data because it permits a data-driven approach. Themes were identified at an explicit level, reducing potential bias, or personal motivation and/or opinion of the researchers. Braun and Clarke (2021) six-phase model of thematic analysis was utilized to ensure thorough data analysis. The six-phase model includes: data familiarization, generation of initial codes, searching for potential over-arching themes, reviewing themes, defining and naming themes, and producing the report.

Data trustworthiness was determined using Forero et al. (2018) four-dimension criteria, adapted from Lincoln and Guba (1985). Criteria include credibility, transferability, dependability and confirmability. Credibility was established through the development of an interview protocol and researcher training in the application of the protocol, triangulation of the data by comparing themes and subthemes to individual transcripts and to existing literature. Regular meetings of the researchers to discuss the data were held. Transferability was achieved by reaching data saturation. Data saturation was reached when no new themes were evident in the dataset. Dependability was achieved by keeping an audit trail of the data and analyses. Data were stored in QSR International’s NVivo-11 data analysis software. Data were coded by four researchers who were consistent in their identification and coding of themes and subthemes. Confirmability was also achieved through these processes.

## Results

### Adult alienated child experiences of reunification

Six themes were identified: (1) catalysts for reunification, (2) factors influencing reunification, (3) alienated child relationships, (4) the role of communication in reunification, (5) alienated child understanding of parental alienation post reunification, and (6) the role of therapy in reunification.

#### Catalysts for reunification

Adult alienated children referred to several catalysts for reunification, within which three subthemes were identified.

##### Readiness of adult alienated child

Five adult alienated children referred to their own readiness as a key factor in seeking to reunify with their targeted parent. Readiness included feeling ready to accept both of their parents after parental alienation.

*What I wanted more than anything in my life was to have a family that was whole. And I was willing to accept my parents for who they were.*

Readiness involved adult alienated children permitting themselves to love their targeted parents after alienation.

*… I think the time I went to my father and I hugged him was when I gave myself permission to love him. I think that’s what reunification is … It’s that permission, that feeling of, ‘I’m allowed to love you.’*

Other adult alienated children described being ready to explore a relationship with their targeted parent in search for a sense of wholeness and resolution.

*I felt like I wanted to find a place where I belonged … I had this parent out there that I had no idea existed … I felt like I was missing part of myself … a small part of a need for me to just … resolve some things.*

##### Personal experiences of divorce or parental alienation

Three adult alienated children described how divorce and alienation from their own children prompted them to reflect on their childhood. They described beginning to question their alienating parent’s behavior and becoming more curious about their own alienation from the perspective of their targeted parent.

*And that’s when I started to realise, ‘okay wait, if [my ex-partner] is going around and easily telling these lies about me, and … people are believing him, I wonder if my mum did that to me about my dad?’*

##### Illness or death of a family member

Two adult alienated children described the illness or death of a family member led to them seeking to reunify with their targeted parent. One adult alienated child described experiencing compassion and love for their targeted parent after the death of a family member, leading them to reconnect. Another adult alienated child reported recognizing the importance of coming together with their targeted parent for support in dealing with the loss of a loved one, rather than grieving alone.

#### Factors influencing reunification

Adult alienated children referred to several factors which they believed influenced reunification with their targeted parent. Factors were classified into three subthemes.

##### Alienating parents

Seven adult alienated children reported that their alienating parent interfered with reunification. For some, interference involved alienating parents actively working against reunification. Interference was also reported involved alienating parents reinforcing negative beliefs or frustrations adult alienated children had about their targeted parents during the reunification process.

*Like just recently I told [my alienating parent] that I was actually contacting [my targeted parent] again and … she doesn’t want to hear it. You can feel it … her entire body is tense if [my targeted parent’s] name’s mentioned … but she’s very happy if I turn around and say, ‘he’s a pain in the arse.’ Then she agrees with me. Then she relaxes. Then she talks about him being a pain in the arse.*

Some adult alienated children referred to the longevity of the alienating parent’s influence on their thinking and behavior post alienation, which can deter reunification attempts. Some reported that their alienating parent rejected them when they resumed contact with the targeted parent. One adult alienated child described how the influence of the alienating parent on reunification continued beyond the alienating parent’s own death.

*I would say my [alienating parent’s] passing away had a negative impact on my relationship with my [targeted parent]. I think [my targeted parent] saw it as a means to open the door because the influence was no longer there, not realising that the influence is still there … I held onto my [alienating parent] probably more tenaciously after her death … than I did before. I think I would have been more open before her passing because it would have allowed me more time to see her flaws and deal with her flaws … and come to terms with the situation.*

##### Other family members and friends

Four adult alienated children stated that other people aside from their alienating parent interfered with reunification. These people included siblings, stepparents, extended family, and friends.

*This is such a nasty, nasty dynamic, this whole thing, because so many people get involved that make it really, really difficult for you to have a relationship with someone that you really care about.*

It should also be noted that family members who were not influenced by the alienating parent’s use of parental alienating behaviors reportedly played a positive role in facilitating reunification.

*… the greater support of the family network was the big contributing factor to just making [reunification] easier and more comfortable … They’d invite me over for dinners and we’d have good family get togethers … so that was a big help.*

##### Physical distance

Two adult alienated children reported that physical distance from their targeted parent interfered with reunification.

#### Adult alienated child relationships

Adult alienated children described the nature of their personal relationships throughout the reunification process. Three subthemes emerged from their descriptions.

##### Connection between adult alienated child and targeted parent

Eight Adult alienated children described how the targeted parent was unfamiliar to them throughout reunification. They described a lack of parent–child attachment bond.

*I don’t love [my targeted parent] … It’s just, there’s nothing there … There’s no bond there at all…And that’s brought about by the fact that … I had no contact with him my entire life until I was already an adult.*

Others described a distant, uncomfortable, or strained relationship.

*[My targeted parent’s] so much like me … and I just love that. And the tenderness that we have, because the tenderness is there but … the physical attachment … you just don’t have the naturalness of it.*

Five adult alienated children reported that they did feel an attachment to their targeted parent. The attachment took the form of a parent–child bond, or a mutual love and understanding.

*I don’t actually have to start from scratch establishing a relationship with my [targeted parent] … it’s not really from scratch because, you know, as buried as it is we do have a bond … because she’s my mother.*

Attachment was also described as noticed similarities in appearance or personality between the adult alienated children and targeted parent.

*I wasn’t like [my alienating parent] and my sister. And then when I met my [targeted parent], and I was like, ‘wow, alright, I’m like my [targeted parent] …’*

##### Reunification requires time and effort

Five adult alienated children explained that reunifying with their TPs required considerable effort from both parties over a protracted period of time. For some, the reunification process spanned decades and involved periods of contact and withdrawal. Adult alienated children referred to effort as needing to be reciprocal and sustained to maintain the relationship.

*I think that human beings being as complicated and complex as they are, nothing is clear cut and this concept that you describe as reunification, you know, it’s kind of like - doesn’t happen easily. It’s something that has to have considered thought and energy put into it. Like all relationships.*

##### Difficulty for adult alienated child to trust

Four adult alienated children reported that they had difficulty trusting others throughout the reunification process. They attributed this to the negative effects of parental alienation and parental alienating behaviors. Four adult alienated children also described that mistrust was internalized and reported having difficulty trusting themselves—their own thoughts, judgments and feelings.

*You’re constantly having to evaluate, ‘should I or shouldn’t I?’, ‘can I or can’t I?’, ‘what does this mean?’, ‘what’s the ulterior motive here?’, ‘is this genuine? Is this not genuine?’ … You know, decades later I have that problem … [My targeted parent] was always very, very genuine, but I had the conflict of, ‘do I or don’t I?’*

#### The role of communication in reunification

Adult alienated children described the role of communication in reunification. This included communication between the adult alienated children and targeted parent and communication between the adult alienated children and alienating parent. Three subthemes were identified.

##### Lack of communication skills to facilitate reunification

Four adult alienated children said they did not have the communication skills to facilitate reunification, and neither did their targeted parent.

*I think [parental alienation] was a topic that everybody found very difficult to discuss. Obviously because it was so fraught with … all the things that had gone on and I think it’s a skill, it’s a learned skill to be able to communicate about topics that are difficult and emotionally charged. And when you haven’t got people around you that are capable of that then everybody just learns to shut down.*

##### Types of communication that facilitate reunification

From all the interviews with adult alienated children, three key communication strategies used by targeted parents appeared to be the most helpful in facilitating successful reunification—objective, persistent and compassionate communication. These strategies appear to be important to building trust.

*But I just think, [my targeted parent] being open, honest, he didn’t shy away from any questions, just that open communication and his gentleness was, he wasn’t defensive, he wasn’t as I would call, or [my alienating parent] said, ‘but, you did this, this and this’, you know, [my targeted parent] would tell his side of it, not demean [my alienating parent] in any way. I think that was probably a huge part that helped me be more trusting of him.*

Persistent (but not unnecessarily frequent) attempts at communication by targeted parents reportedly helped to facilitate reunification. Forms of attempted communication included phone calls, messages, letters, and attendance at events.

*I think parents that reach out every day, and text every day, it’s a bad move. It’s very easy to become numb to it. It’s like no big deal. I think the fact that [my targeted parent] did reach out on occasion, even though I didn’t like him, and I was angry at him, I think that he couldn’t be forgotten because he was showing up.*

Some adult alienated children advised that they were not aware of their targeted parent’s attempts at communication, due to the influence of their alienating parent. However, learning about these attempts through the process of reunification was also helpful in facilitating reunification.

*[My targeted parent] handed me … a stack … probably 10 inches of paperwork, and he just gave it to me, I didn’t ask for it, he was just like, you know, ‘here is everything I’ve kept over the years.’ He did go to my high school graduation, I just didn’t know he was there … I was a sobbing mess by the time I got done with reading through all that stuff. So, I think it was that he wasn’t telling me what I should get out of that paperwork.*

##### Types of communication that hinder reunification

Adult alienated children made note of three communication styles used by themselves, their targeted parents and alienating parents that hindered reunification—avoidant or reactive, past-focused, and confrontational communication. Five adult alienated children reported that if targeted parent or alienating parent were avoidant or reactive to conversation around alienation, it hindered the adult alienated children’s ability to consolidate their relationships.

*I was questioning things, and sometimes I could talk to my [alienating parent] or my [targeted parent]. I could talk to them about certain things but quite often, I could ask them something and it would set them off and they’d go into a fury. Either my mother or my father, both of them, it would hit a nerve. So, I very quickly learnt that it was best to leave those subjects alone.*

Five adult alienated children also referenced past-focused communication by their targeted parents as being a hinderance to reunification. Adult alienated children found these conversations emotionally challenging and they contributed to their own sense of mistrust and defensiveness.

*He’d [targeted parent] say ‘oh, do you remember that house I used to live in, and you used to come and would swing on the swing and this tree’ and he can remember it all vividly and basically, I called him a liar because I couldn’t remember anything.*

Two adult alienated children reported being confrontational in their approach to communicating with their targeted parents, which created more distance in their relationship.

*I had no empathy when I met with my [targeted parent] … I was just basically saying, ‘well why didn’t you call me? How come you never sent me any birthday gifts? Why didn’t …’ And I hammered her with all these things and um, she said ‘you’re exactly like your father’.*

#### Adult alienated children’s understanding of alienation post reunification

Adult alienated children shared their understanding of parental alienation after having reunified with their targeted parents. Reflections were divided into five sub-themes.

##### Withholding blame for alienation

Seven adult alienated children stated that they did not blame either parent for their alienation after reunification. Instead, they accepted both parents for who they are and could see both perspectives. They no longer felt obliged to choose between them.

*As an adult, I realise that people are flawed, and my parents are flawed. And it’s not that my mum was all good and my dad was all bad. People are complicated.*

##### Lack of ideal parental relationships

Five adult alienated children acknowledged that the nature of their relationships with their parents were not their version of ideal.

*But I have to say, on hindsight, I mean, I didn’t have as good a relationship with my dad as I would have liked, and I haven’t had as good a relationship with my mother as I would have liked. It’s been turbulent for me with both my parents.*

##### Sympathy for parents

Four adult alienated children expressed sympathy for one or both of their parents. They described that post reunification they were able to appreciate the negative effects of alienation on their targeted parent. One expressed sadness for their alienating parent, recognizing that their behavior was a product of their own upbringing.

*I probably have more sadness for [my parents] than I do for myself … especially my [alienating parent] now knowing what she went through when she was growing up, and that she was just merely a product of … what had been done to her. I think both my parents could have had happier lives, but … this parental alienation is very powerful. And it’s very damaging and it affects a lot of people.*

#### Reflections on alienating parent behaviors

Three adult alienated children reflected on parental alienating behaviors. They discussed being turned against their targeted parent and not having had the opportunity to develop a relationship with them.

*The issue that I’ve always had with my [alienating parent] as an adult and on reflection of what the choices were that were made, was that I wasn’t considered in the situation. My needs as the child and my needs as having or needing to have a relationship with my [targeted parent] were not considered. So, [my alienating parent] didn’t see the importance or the value of me needing to have a relationship with my [targeted parent].*

##### Guilt and shame

Three adult alienated children expressed feeling some level of guilt and shame.

*I would say that I felt guilty. I’m not sure that was mine to own, but I felt guilty that [my targeted parent] had to experience [parental alienating behaviours].*

#### The role of therapy in reunification

Three adult alienated children reported having attended therapy to discuss reunification. Two adult alienated children described benefiting from therapy because they learnt how to understand their own feeling and how to view their parents as equals.

*I feel very grateful that I had the therapist I did … she was the one who helped me to see … my parents equally. I had always, you know, my [alienating parent] was up on this pedestal up here and my [targeted parent] had been down here, and she kind of brought them equal.*

### Targeted parent experiences of reunification

Three themes were identified in the analysis of 12 TP interviews including: (1) what is reunification, (2) factors impacting reunification, and (3) life post-reunification. Several sub-themes and sub-subthemes were identified.

#### What is reunification?

TP definitions of reunification were varied. Four sub-themes relating to four aspects of reunification were identified.

##### Reunification is subjective

All targeted parents considered themselves to be reunified with their adult alienated children, however the type of relationship and level of contact with their adult alienated children varied. In some cases, the adult alienated children resided with their targeted parent full-time, some lived with their alienating parent, and some lived alone. Contact between the targeted parent and adult alienated child ranged from daily to occasional contact. Some targeted parents had been reunified with their adult alienated children for years, whereas others had only been reunified for several weeks.

##### The catalyst event

All targeted parents reported that reunification was a decision that their adult alienated children made following a catalyst event. Two targeted parents reported that people in the adult alienated child’s life prompted the adult alienated child to make contact. The prompting appeared to take the form of the person asking curious questions about their relationship, or lack thereof, with the targeted parent.

*You just never know, it could be the flick of a switch, that something can happen and it’s a trigger. The trigger was my son’s [alienated adult child’s] mate. He said, ‘what’s the deal with your mum, you never talk about her?’ And that’s what triggered him … And he’s like, ‘well why don’t you go see her, why don’t you reach out to her?’*

For another targeted parent the catalyst for reunification was that parent being diagnosed with a degenerative disease. Other targeted parents speculated that it was the adult alienated child’s age and increased independence which prompted their contact.

##### Reconciling with extended family and friends

Five out of 12 targeted parents discussed how the adult alienated child reconnecting with extended family and friends was part of the reunification process. Targeted parents reported that alienation extended to family and friends and so did reunification.

*She [adult alienated child] saw my mother for the first time in more than three years on Saturday. And she’s seen her aunt. So gradually she’s starting to see other people from our family and making future plans to them.*

##### Reunification is a process

All targeted parents described spending significant time and effort trying to repair the fractured relationship with their adult alienated child prior to successfully reunifying. They described how letters, cards, gifts, emails, text messages, and phone calls were consistently unanswered for years before contact became reciprocal. One targeted parent reported driving 2 h every Christmas, Easter, and birthday over the course of a decade to spend 5 min with her child and hand over gifts, before driving 2 h home.

Some targeted parents spent years in the family court, accumulating significant debt in the process, to no avail. Others described their child as being abusive toward them during the reunification process. For seven targeted parents, reunification after a catalyst event was immediate. For five targeted parents, reunification was a protracted process characterized by periods of reconnection and withdrawal by the adult alienated child.

*It’s been a real rollercoaster, and she’s come back I think about five times and then gone again, so it-it’s been a process. It’s not just suddenly, ‘Mom, I’m sorry’, you know, ‘I really didn’t mean to, I love you’ then we’re all ok. It hasn’t been like that at all. It’s been really, like, one step forward two steps back. For years … it’s been a good six or seven years…*

Some targeted parents speculated that the withdrawal occurred because they challenged their adult alienated child in some way, such as enforcing boundaries, or because they would not give in to their adult alienated child’s demands (e.g., to purchase a new car). One targeted parent said their adult alienated child withdrew because their child said they missed their siblings who still lived with the alienating parent. In this instance, the targeted parent said the alienating parent prevented the adult alienated child from communicating with their siblings while they were maintaining contact with the targeted parent.

#### Factors impacting reunification

All targeted parents identified factors that impacted the reunification process. Two sub-themes emerged.

##### Factors that helped reunification

Ten targeted parents said support from others helped to facilitate reunification. Others included joining a parental alienation support group, speaking to other targeted parents, seeking counseling, and receiving emotional and practical assistance from friends and family members.

*The other things that really helped were seeing a family therapist who specialises in this area and going to parental alienation support groups… It makes a big difference to talk to people who have been there.*

Nine targeted parents described learning about parental alienation, parental alienating behaviors and reunification. They reported that reading about parental alienating behaviors allowed them to understand what was happening to them and why their child had rejected them. This knowledge was considered an essential part of maintaining their own mental health during the alienation and subsequent reunification process.

*Oh, in the early time I had no idea about parental alienation and so it was hell, because I thought ‘what is going on’ … you start going crazy because you had this fantastic bond with your kid, and then … it just explodes and you think, this is impossible!…you read up on parental alienation and it’s like a light-switch and you think ‘thank god, there’s something out there which explains this’ … That was a real life-saving moment for me.*

For some targeted parents, meeting other targeted parents, recently reunified targeted parents and other adult alienated children provided them with insight into a child’s perspective of parental alienation. Some targeted parents said this insight was instrumental in shaping how they coped with the alienation and managed the reunification process.

Eight targeted parents said starting afresh with their adult alienated children was helpful in the reunification process. This involved maintaining a focus on the present rather than dwelling on the past. Targeted parents also said rebuilding the relationship with their child at their child’s pace, providing a happy and loving home environment, avoiding talking about the past, and making a conscious effort to display at least neutral feelings toward the alienating parent all helped the reunification process. They also said it was important to resist the temptation to disclose details of their experience during the alienation because this creates conflict and can result in the adult alienated child withdrawing from them. All targeted parents noted that it was important to move on from the alienation and accept that their child may be unable to provide them with explanations for their rejection.

*I was over the whole thing. I wasn’t angry at anybody … Reconnecting with my son was not about me. It wasn’t about, you know, getting an apology from him. It wasn’t about getting any kind of validation that … I wasn’t such a horrible human being. It wasn’t anything to do with me. It was just solely about him and the present and the future … I remember in the beginning he used to call me by my first name and, you know … I had a choice to make … do I get annoyed and angry and upset because he’s calling me by my first name? Or do I just let it go? I decided to let it go … He hasn’t talked to me for twelve years, now all of a sudden he’s talking to me. It’s like am I going to get hung up over that fact that he’s calling me by my first name instead of saying ‘Dad’? No.*

##### Factors that hindered reunification

Seven targeted parents observed that their adult alienated child’s mental health hindered reunification. They described their children as being emotionally dysregulated, fearful, anxious, aggressive, controlling and/or submissive. Targeted parents attributed this to the unhealthy and harmful relationship adult alienated children had with their alienating parent.

*… he (adult alienated child) would put his hands on his head, and he’d say, ‘I don’t know why I keep doing this,’ he said, ‘I just don’t know, I just get this feeling of being angry and I just want to blame you.’*

Ten targeted parents described how the “system,” namely family law courts, was ineffective in facilitating reunification. The targeted parents blamed the family law courts for failing to intervene and allowing the alienation to progress over many years. They explained that practitioners within family law courts are inadequately educated on parental alienating behaviors and consequently, the needs of the alienated child go unseen.

*Once they’re over the age of twelve it’s so-called children’s wishes. So even though the courts knew that her father had got her to make those gross allegations, there were no consequences and he refused to do any court ordered family therapy.*

Targeted parents reported that the process of going through the family law court to reunify with their child, facing the alienating parent and defending themselves against false allegations made by the alienating parent had significant impacts on their mental health.

*The family law court … that was a horrible, horrible, horrible experience … I really did want to kill myself going through that … I think I’ve got PTSD from that … I try and … not to talk about that.*

Seven targeted parents were still alienated from at least one of their children, which hindered the reunification process with the child who had initiated reunification. These targeted parents described trying to cope with being a parent to the reunified child, while grieving for their other alienated child/ren.

*People think that because I have one child back that I am lucky and everything is great, but you know, it’s like, I’ve got two children. And I still grieve the same way I grieved when I didn’t have both of them in my life.*

Five targeted parents reported that their child’s alienating parent continued to purposefully engage in parental alienating behaviors, hindering reunification.

#### Life after reunification

TPs provided insight into their lives post reunification. Four sub-themes were identified.

##### Feelings toward the alienating parent

All targeted parents expressed mixed feelings toward the alienating parent. These feelings included apathy, pity, fear, anger and empathy. Some participants reported disbelief that the person they had chosen to have a child with could inflict such harm upon them.

##### We do not speak of the alienation

Nine targeted parents reported that they did not speak about the period of alienation with their adult alienated child. They felt trepidation toward broaching the topic. This was reportedly because the adult alienated child continued to defend their alienating parent. These targeted parents stated that if their adult alienated child expressed a desire to discuss the alienation, they would be willing.

*It’s kind of strange. Sometimes it’s like the elephant in the room … One of the decisions I made early on if like if he wanted to talk about it, I was willing to talk about it, but if he didn’t want to talk about it, you know, we weren’t going to talk about it.*

##### The negative impacts of reunification

Six targeted parents shared that reunification had negatively impacted their mental health. For some, reunification reminded them of how much they had missed out on in their children’s lives. They described the physical consequences of enduring years of distress and grief associated with parental alienation.

*There’s been a lot of impact on me … it’s been incredibly physically demanding in terms of interrupted sleep and sort of the continual emotional drain of dealing with an extremely traumatised child … the whole process has felt a bit like being fried to death with my own cortisol.*

Five targeted parents also referenced the negative financial consequences of reunification. They described spending years in court trying to have a relationship with their children at great financial cost. Some described needing to cease work to care for their adult alienated child full-time, due to the child’s experience of emotional and behavioral difficulties as consequences of parental alienation.

*Like you know, some of the big picture stuff is that I’ve had to sell the only home I’ve ever owned. I’ve spent my superannuation … you know I’m kind of basically destitute at retirement age and that’s basically because of having spent the last decade trying to protect my kids and spending vast amounts of money.*

##### The positive impact of reunification

Seven targeted parents referred to the positive impact reunification had on their lives. They said meaning had been restored in their lives.

*I feel more complete. I’ve got my sense of purpose back. I can mother him, cook for him and do things. Now it’s not just the cat and dog, I’ve got my son … it’s part of having that purpose again. Feeling like I’m a mum again.’*

## Discussion

The study explored the experience of voluntary reunification post alienation from the perspectives of adult alienated children and targeted parents. The findings provide further insights into the process of reunification, how it occurs, and how it can be supported.

### Defining successful voluntary reunification as a child-driven process

Voluntary reunification is defined in the literature as a process that occurs when an adult alienated child actively seeks to restore a relationship with their targeted parent without a court mandate or reunification therapy (Darnall and Steinberg, 2008b). In the current study, all targeted parents reported that reunification was initiated by their adult alienated child, and it was a child driven process. Targeted parent and adult alienated child participants described reunification as a protracted process, sometimes across many years with phases of contact and withdrawal by adult alienated children. This is consistent with Rand and Rand (2006) who described reunification as a fragile process. The apparent approach-withdraw cycle seen in the experiences of participants in the current study can be understood using attachment theory.

When parental alienating behaviors result in the eradication of the targeted parent from the alienated child’s life, this attachment bond is damaged along with the child’s attachment system. The alienated child’s attachment system may be further impacted by the nature of their relationship with the alienating parent. If this relationship is unstable in some way, the child is then unable to experience a secure base from the other attachment figure (Haines et al., 2020). Consequently, adult alienated children may be wary of the targeted parent until regular contact recommences, there is an opportunity for repair of the relationship and trust is built (Haines et al., 2020). For the attachment bond to be strengthened the adult child needs to have warm, involved, and continuous contact from the targeted parent. The approach-withdraw process of reunification could be the way in which the adult alienated child begins to explore their relationship with their targeted parent. It may be a process of testing the waters, seeing if they are safe, withdrawing when overwhelmed and unsure, and reapproaching when ready. The need for the targeted parent to be a secure base and a source of reliable comfort is essential during this process (Haines et al., 2020). The current study’s findings suggest that adult alienated children need to be in control of the reunification process for it to be successful. This may be because much of the parental alienation process is outside of their control. These findings mirror literature on the importance of empowering survivors of child abuse to take the lead in their own recovery (van Loon and Kralik, 2005).

### Frequency of contact, quality and depth of relationship

The current study found that targeted parent–child relationships during reunification varied in the amount, quality, and depth of contact. Relationships were described on a spectrum of unfamiliar, distant, lacking a bond, and having difficulties with trust, to feeling degrees of connectedness and mutual love. Darnall and Steinberg (2008a) similarly reported that the targeted parent–child relationship during the reunification process can vary in intensity over time.

It is suspected that fluctuation may also be due to factors including the age of the alienated child at the commencement of parental alienation. If a child is very young, they may not have the opportunity to solidify an attachment bond with the targeted parent prior to being alienated from them. Other possible factors impacting reunification include the severity of the parental alienating behaviors and readiness of the child to reunify. Further, the psychological functioning of the adult alienated child and targeted parent during the reunification process may impact on reunification outcomes. Further research is required to explore these factors to determine causation.

### Readiness of the adult alienated child

Consistent with previous research (Rand and Rand, 2006; Baker, 2007; Darnall and Steinberg, 2008a,b), there are a number of possible catalysts for reunification. The most consistent trigger for a resumption of contact was the adult alienated child’s readiness to do so. Readiness centered on a willingness to accept and love both their parents following parental alienation, and to a explore a relationship with their targeted parent in search of a resolution to the alienation. Intrinsic changes within the adult alienated child (e.g., maturation or reframing inaccurate beliefs about the targeted parent) may lead to reconnection (Rand and Rand, 2006; Baker, 2007; Darnall and Steinberg, 2008a,b).

### Factors that influence reunification

Participants identified several factors that influence reunification. Factors that hindered reunification included the ongoing influence of parental alienating behaviors, the adult alienated child’s capacity to trust the targeted parent, geographical distance between the adult alienated child and targeted parent, and the way in which the targeted parent behaved during the reunification process.

Findings illustrate the pervasive nature of parental alienating behaviors, and that parental alienation is not just an experience limited to childhood. Parental alienating behaviors continue to impact the child well into adulthood affecting their ability to re-connect and consolidate relationships with their targeted parent. To assume the child will simply reconnect with the targeted parent when they are an adult is erroneous (Templer et al., 2017). The influence of parental alienating behaviors is enduring and traumatic, and the subsequent reunification process is complex (Haines et al., 2020; Verhaar et al., 2022).

These findings can be understood in the context of family violence. Abusive behaviors, particularly coercive control, are pervasive (Candela, 2016). If parental alienating behaviors are considered abusive behaviors, it is unlikely that these behaviors will abate simply because the child has become an adult. Perpetrators of family violence do not easily surrender their power and control (Candela, 2016).

Consistent with the experience of child abuse survivors, some of the adult alienated child participants in this study described having difficulty trusting themselves and others during the reunification process. These trust difficulties reportedly slowed the reunification process. Other studies have also found lack of trust in self and others may be a long-term consequence of being exposed to parental alienating behaviors in childhood (Baker, 2007; Bentley and Matthewson, 2020; Verhaar et al., 2022). This lack of trust may be due to being told by a parent that their other parent is unavailable, unloving and/or threatening in some way contrary to their own experiences of their other parent. Losing that attachment figure and remaining in the custody of an attachment figure who is using coercive control makes it difficult for the child to not only trust others, but to trust their own judgment and perceptions (Harman et al., 2021; Verhaar et al., 2022).

For both the adult alienated child and the targeted parent, exposure to parental alienating behaviors and parental alienation are traumatic experiences (Haines et al., 2020; Verhaar et al., 2022). This study showed that the reunification process may be influenced by the psychological functioning of the targeted parent and adult alienated child. The results suggest that, while the reunification process needs to be largely driven by the adult alienated child, how the targeted parent is functioning and able to cope with the approach and withdraw pattern of that process is important. A person who has experienced psychological trauma can experience hyper and/or hypoarousal. In these states, a person’s body detects threat and is primed for survival, which can prevent social engagement. If a traumatized person does not have the means to regulate themselves or have a stable base with which to co-regulate, dysregulation may persist, or become heightened (van der Kolk, 2003). If the adult alienated child and targeted parent are unable to manage their trauma reactions their capacity to build a trusting relationship will be hindered. The results also suggest that if the targeted parent can provide a secure base and the adult alienated child is ready and able to receive it, trust and co-regulation may be possible.

The targeted parent’s own coping may influence their ability to effectively communicate with their adult alienated children. Communication was found to be an important factor in the reunification process. It appears if targeted parents are avoidant, reactive, past-focused, and confrontational in their communication with the adult alienated child, trust cannot be built, and the reunification process is hindered. This is because adult alienated children may find conversations of this nature emotionally challenging, dysregulating, and destabilizing (Bentley and Matthewson, 2020; Haines et al., 2020). This is consistent with Warshak (2010), who recommended that targeted parents “strike while the iron is cold.” This means it is important for targeted parents to resist the urge to have or persist with confronting topics of conversations with their adult alienated children when the adult alienated child is ready and not when they are having strong emotional reactions.

Additionally, the current study also revealed that other people aside from the alienating parent (e.g., siblings, stepparents, extended family, and friends) interfered with the reunification process. This is consistent with Haines et al. (2020) who highlighted that families consist of more than parent–child triads and extended family can indeed be involved in facilitating parental alienation, but they can also be important in facilitating reunification.

Factors that helped reunification included support from family, friends, peers, and mental health professionals, increased education and understanding of parental alienation, objective, present-focused and persistent communication, and a willingness for some to “start afresh.” The findings indicate that targeted parents and adult alienated children benefit from understanding parental alienation, parental alienating behaviors and their own experience of these when approaching reunification. Participants in this study described how knowledge and the support of others helped them to heal from their traumatic experience so they were able to reconnect with another person, who had also had a traumatic experience.

### Impacts of reunification

Adult alienated children and targeted parents described the consequences of reunification on their lives. Adult alienated children reported developing a sense of acceptance and understanding of their circumstances and the people involved. They learnt to withhold blame for the alienation and have sympathy for both parents. They learnt to have healthier critical thinking skills and recognize that their relationships with their parents are complex. Adult alienated children brought guilt and shame into the reunification process that needs to be explored further in future research. Adult alienated children’s experience of guilt and shame should not be overlooked if they seek therapeutic support before, during or after reunification.

Some targeted parents described their lives as being enriched and regaining meaning through re-establishing a relationship with their child. This finding is consistent with posttraumatic growth theory, which proposes that some people can make meaningful and positive changes as a result of traumatic experiences (see Calhoun et al., 2010; Tedeschi et al., 2018). Other participants described having difficulty parenting their adult alienated children post-reunification due to their mental and physical health and financial situations. Targeted parents in the current study needed to manage their own mental health during reunification while supporting their adult alienated children who were often equally as traumatized. They needed to learn how to parent a traumatized adult who is very different to the child they last saw before being alienated from them. These findings suggest that therapeutic support during the reunification process should be considered by the adult alienated child and targeted parent. It is important that the adult alienated child and targeted parent have available to them a wealth of healthy self-care strategies and the capacity to implement healthy boundaries.

### Clinical implications

For practitioners to support families during voluntary reunification, it is important that they have a good understanding of parental alienating behaviors, parental alienation, and their impact. The current study identifies specific areas on which intervention and support frameworks for reunifying families can be based. Based on the findings and consistent with Haines et al. (2020), it is recommended that adult alienated children and targeted parents seek therapeutic and social support so they can start to heal from their trauma, address unresolved grief, shame, and guilt, and learn how to establish healthy patterns of communication and interaction. Therapeutic approaches need to be trauma informed and may include trauma and grief processing, family of origin work to understand the intergenerational transmission of trauma to reduce blame, acceptance practices, cognitive restructuring, assertiveness training, coping skills training and parenting support.

### Limitations and directions for future research

The size of the current study’s sample was relatively small. Nonetheless, it was robust due to the richness of data and having met data saturation. It is recommended that future research include data from a larger sample involving qualitative and quantitative methods to increase generalizability of results and to further explore casual relationships between the variables that might predict, mediate and/or moderate reunification outcomes.

The qualitative data provided by participants in this study are considered by the researchers to be a true interpretation of their experiences, however, it is important to acknowledge that adult recall of past experiences is subjected to self-report bias. Memory of past events can be influenced by the passage of time, suggestion, and personal biases (Kensinger, 2009). This is a limitation inherent in qualitative research, however, the richness of the data obtained in this study is valuable and insightful. This is because adults can provide rich and detailed information about their memories, emotions, and perception of their experiences regardless of their age (Kirkegaard Thomsen and Brinkmann, 2009). Future research may aim to obtain information from collateral sources or using a longitudinal design to enhance the robustness of the research.

In terms of vulnerability to parental alienation, some research suggests that there are no significant differences between male and female adult alienated children (Baker and Darnall, 2006; Baker, 2010a). Other research indicates that females may be more vulnerable (Balmer et al., 2018). Similarly, the literature suggests an equal prevalence of targeted mothers and fathers (Balmer et al., 2018). Based on limited literature, it is unclear as to whether these distributions also apply in cases of voluntary reunification. The current study is based on a majority female sample of adult alienated children and targeted parents, therefore, it may not accurately capture the voluntary reunification experience of male adult alienated children and targeted parents and the experience of people who are gender diverse. It is recommended that future research include more gender-varied samples. This will create opportunities for the assessment of gender differences and recommendations for tailored intervention.

In the current study, the length of time adult alienated children were alienated from their targeted parents and the length of time since reunification were estimated in collaboration with participants. This is because some adult alienated children had difficulty quantifying their alienation and reunification experiences. Demographic information may have been impacted by the participants’ memories of past events. This may have been the result of some participants needing to protect themselves from past traumatic events (Goodman et al., 2010), the outcome of parental alienating behaviors (Baker, 2005a,b), and/or a combination of both factors. The influence of recalling past traumatic events cannot be ignored as a possible limitation of the study’s findings. It is recommended that future research be conducted longitudinally, following the trajectory of families post separation and beyond to limit data reliance on past recall. It is also suggested that future research include collecting information from collateral sources and information from members of the wider family system.

A limitation inherent in all qualitative research is the potential for researcher bias. Attempts were made to safeguard against this by approaching data analysis from an inductive data-driven stance and by using multiple coders to ensure inter-rater reliability. To enhance generalizability of future research, it is recommended that studies use a multimodal approach. Despite these limitations, the current study offered unique insight into the voluntary reunification process following parental alienation.

“*Rebuilding the parent-child relationship when the child is an adult requires commitment and patience. The process is fragile, and vulnerable to breaking down. The desire of parent and child to reconcile must be strong enough to work through the powerful emotions which stand in the way, such as fear, anger, and guilt*.” (Rand and Rand, 2006, p. 164).

## Data availability statement

The raw data supporting the conclusions of this article will be made available by the authors, without undue reservation.

## Ethics statement

This study was reviewed and approved by the University of Tasmania Social Sciences Human Research Ethics Committee. The participants provided their written informed consent to participate in this study.

## Author contributions

MM coordinated and oversaw all aspects of the research. JB, JH, and SW conducted the interviews. MM, JB, JH, and SW conducted the data analyses. All authors contributed to writing the manuscript.

## Funding

This work was funded by the University of Tasmania.

## Conflict of interest

The authors declare that the research was conducted in the absence of any commercial or financial relationships that could be construed as a potential conflict of interest.

## Publisher’s note

All claims expressed in this article are solely those of the authors and do not necessarily represent those of their affiliated organizations, or those of the publisher, the editors and the reviewers. Any product that may be evaluated in this article, or claim that may be made by its manufacturer, is not guaranteed or endorsed by the publisher.
